# Supplementary material for: Uses of Inflammatory Markers for Differentiation of Intrahepatic Mass-Forming Cholangiocarcinoma from Liver Abscess: Case-Control Study
Source: J Clin Med. 2020 Oct 1;9(10):3194. doi: 10.3390/jcm9103194 (PMC7599997; doi:10.3390/jcm9103194)
Supplement: Supplementary file 1 [file jcm-09-03194-s001.pdf]

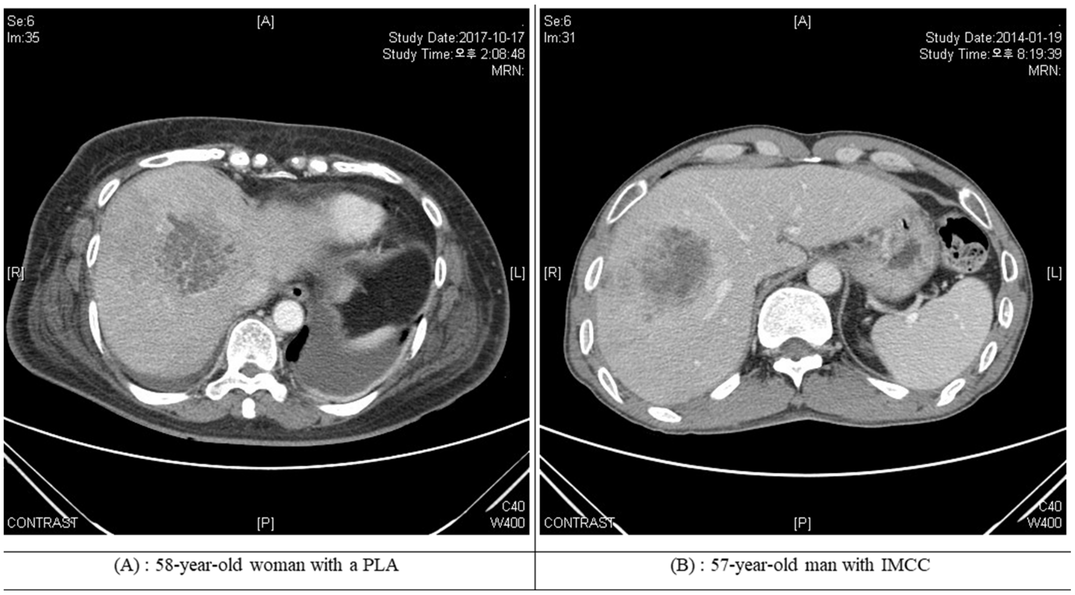

(A) : 58-year-old woman with a PLA	(B) : 57-year-old man with IMCC

Figure 1. Similar CT images with difficult to distinguish PLA and IMCC. (**A**) 58 year old woman with a PLA. (**B**) 57 year old man with IMCC. CT: Computed tomography; PLA: pyogenic liver abscess; IMCC: intrahepatic mass-forming cholangiocarcinoma.
